# Ultrashort echo time MRI of the lung in children and adolescents: comparison with non-enhanced computed tomography and standard post-contrast T1w MRI sequences

**DOI:** 10.1007/s00330-021-08236-7

**Published:** 2021-10-20

**Authors:** Diane M. Renz, Karl-Heinz Herrmann, Martin Kraemer, Joachim Boettcher, Matthias Waginger, Paul-Christian Krueger, Alexander Pfeil, Florian Streitparth, Karim Kentouche, Bernd Gruhn, Jochen G. Mainz, Martin Stenzel, Ulf K. Teichgraeber, Juergen R. Reichenbach, Hans-Joachim Mentzel

**Affiliations:** 1 Department of Paediatric Radiology, Institute of Diagnostic and Interventional Radiology, Hannover Medical School, Carl-Neuberg-Str. 1, 30625 Hannover, Germany; 2 Medical Physics Group, Institute of Diagnostic and Interventional Radiology, Jena University Hospital, Friedrich-Schiller-University, Jena, Germany; 3 Friedrich-Schiller-University, Jena, Germany; 4 Department of Paediatric Radiology, Institute of Diagnostic and Interventional Radiology, Jena University Hospital, Friedrich-Schiller-University, Jena, Germany; 5 Department of Internal Medicine III, Jena University Hospital, Friedrich-Schiller-University, Jena, Germany; 6 Department of Radiology, University Hospital Munich, Ludwig-Maximilians-University, Munich, Germany; 7 Department of Paediatrics, Jena University Hospital, Friedrich-Schiller-University, Jena, Germany; 8 Department of Paediatric Pulmonology and Cystic Fibrosis, Brandenburg Medical School, University Hospital, Brandenburg, Germany; 9 Department of Paediatric Radiology, Children´s Hospital, Cologne, Germany; 10 Institute of Diagnostic and Interventional Radiology, Jena University Hospital, Friedrich-Schiller-University, Jena, Germany

**Keywords:** Magnetic resonance imaging, Tomography, x-ray computed, Lung, Child, Neoplasms

## Abstract

**Objectives:**

To compare the diagnostic value of ultrashort echo time (UTE) magnetic resonance imaging (MRI) for the lung versus the gold standard computed tomography (CT) and two T1-weighted MRI sequences in children.

**Methods:**

Twenty-three patients with proven oncologic disease (14 male, 9 female; mean age 9.0 + / − 5.4 years) received 35 low-dose CT and MRI examinations of the lung. The MRI protocol (1.5-T) included the following post-contrast sequences: two-dimensional (2D) incoherent gradient echo (GRE; acquisition with breath-hold), 3D volume interpolated GRE (breath-hold), and 3D high-resolution radial UTE sequences (performed during free-breathing). Images were evaluated by considering image quality as well as distinct diagnosis of pulmonary nodules and parenchymal areal opacities with consideration of sizes and characterisations.

**Results:**

The UTE technique showed significantly higher overall image quality, better sharpness, and fewer artefacts than both other sequences. On CT, 110 pulmonary nodules with a mean diameter of 4.9 + / − 2.9 mm were detected. UTE imaging resulted in a significantly higher detection rate compared to both other sequences (*p* < 0.01): 76.4% (84 of 110 nodules) for UTE versus 60.9% (67 of 110) for incoherent GRE and 62.7% (69 of 110) for volume interpolated GRE sequences. The detection of parenchymal areal opacities by the UTE technique was also significantly higher with a rate of 93.3% (42 of 45 opacities) versus 77.8% (35 of 45) for 2D GRE and 80.0% (36 of 45) for 3D GRE sequences (*p* < 0.05).

**Conclusion:**

The UTE technique for lung MRI is favourable in children with generally high diagnostic performance compared to standard T1-weighted sequences as well as CT.

**Key Points**

• *Due to the possible acquisition during free-breathing of the patients, the UTE MRI sequence for the lung is favourable in children.*

• *The UTE technique reaches higher overall image quality, better sharpness, and lower artefacts, but not higher contrast compared to standard post-contrast T1-weighted sequences.*

• *In comparison to the gold standard chest CT, the detection rate of small pulmonary nodules small nodules* ≤ *4 mm and subtle parenchymal areal opacities is higher with the UTE imaging than standard T1-weighted sequences.*

**Supplementary Information:**

The online version contains supplementary material available at 10.1007/s00330-021-08236-7.

## Introduction

Magnetic resonance imaging (MRI) of the lung is increasingly established in children and adolescents [1–7]. According to the recently published systematic review of Hirsch et al. [2], lung MRI enables to replace up to 90% of paediatric chest computed tomography (CT) examinations. In contrast to adults, paediatric primary lung tumours are rare with the majority of pulmonary malignancies being of metastatic origin [2, 4, 8, 9]. However, the detection of subtle pulmonary pathologies, particularly tiny nodules, by lung MRI is still challenging [10–13]. Sub-centimetre sized pulmonary nodules are mostly benign in general paediatric populations [14–16]. Otherwise, even small pulmonary nodules can be malignant in children with proven malignancies, as many paediatric tumours can develop metastases in the lungs [4, 17].

Because children are more sensitive to negative effects of X-ray exposure than adults, one main benefit of lung MRI is the absence of any radiation exposure [2–5]. Further advantages are the possibility to visualise pathologies by using differently weighted sequences and to provide morphologic as well as functional information [2–5, 18, 19]. The main issues of lung MRI are low proton density of lung parenchyma and strong magnetic susceptibility effects resulting in short T2 and T2* relaxation times [10, 18–22]. Further challenges are motion artefacts due to patient movement, cardiac pulsations or respiration, which are particularly problematic for younger children who have high cardiac pulse and respiratory rates as well as low capabilities to cooperate during breath-hold manoeuvres [2, 4, 23]. T2-weighted lung MRI sequences can be acquired by breath-hold manoeuvres and/ or by respiratory navigator gating; this respiratory navigator gating technique cannot be sufficiently used in T1-weighted sequences due to their short echo (TE) and repetition times (TR) [18, 19].

Standard T1-weighted lung  MRI sequences, currently mainly used in clinical routine, are acquired during breath-hold technique with common slice thicknesses of ≥ 2.5 mm [18, 24, 25]. The recently developed ultrashort echo time (UTE) sequences have very short TE on the order of < 0.1 ms combined with rapid acquisition due to short TR, which makes them suitable to acquire signals from tissues with short T2/T2* relaxation times [18, 26–29]. Three-dimensional (3D) UTE techniques allow isotropic resolutions with slice thicknesses around 1 mm and, due to their numerous excitations, can be acquired during free-breathing of the patients [26–31]. As far as we know, this is the first study, which has performed a detailed analysis of a 3D UTE sequence of the lung with isotropic voxels in a paediatric oncologic population, including an objective and subjective imaging assessment. The aim of this prospective study was to compare the UTE technique to chest CT, which is still the gold standard, and to two T1-weighted breath-hold sequences (2D incoherent and 3D volume interpolated gradient echo (GRE)), which are currently the most performed standard T1-weighted sequences in clinical routine. The focus of this investigation was set on the diagnosis of challenging, subtle, pulmonary pathologies, including small nodules and ground-glass opacities.

## Materials and methods

### Study design and patients

The study was planned and performed in accordance with the ethical guidelines of the Declaration of Helsinki and was approved by the local ethics committee (institutional review board number 4562–10/15). The custodians and, in the case of competence (comprehension and intelligence), also the children and adolescents gave consent before participating in the study. This prospective investigation was initiated by the investigators and designed as an intraindividual comparison between three T1-weighted post-contrast MRI sequences and non-enhanced chest CT examinations at one single study centre.

Children and adolescents with proven oncologic disease, who had been referred to clinically indicated MRI examinations with an intravenous application of MR contrast medium as well as to an unenhanced low-dose chest CT, were included. Main clinical indications for the MRI examinations were the diagnosis of lymph nodes and bone metastases. Further inclusion criteria were the performance of the MRI and the CT scans within a maximum of 10 days to provide a reliable comparison, a health status of the patients, which enables to slightly prolong the MRI examinations for study purposes and the possibility to acquire images during breath-hold of the patients. Exclusion criteria were contraindications against MRI and/or MR contrast media, chronic kidney disease ≥ stage 3 (glomerular filtration rate < 60 mL min^−1^ (1.73 m^2^)^−1^), and application of any contrast medium within 24 h before the MRI and/or CT examinations.

Twenty-three patients (14 male, 9 female) with 35 CT and MRI examinations fulfilled the inclusion criteria. The patients suffered from the following oncologic diseases: nephroblastoma (*n* = 8), acute lymphoblastic leukaemia (*n *= 4), Ewing’s sarcoma (*n* = 3), osteosarcoma (*n* = 2), Hodgkin’s lymphoma (*n* = 2), rhabdomyosarcoma (*n* = 2), thyroid carcinoma (*n* = 1), and mediastinal seminoma (*n* = 1). The mean age of the patients was 9.0 years (standard deviation, SD, 5.4 years; range 1–17 years). Fourteen of the 35 CT and MRI examinations (40.0%) were acquired under general anaesthesia of the patients, the remaining 21 CT and MRI scans (60.0%) without any sedation or anaesthesia.

### CT and MRI protocol

All patients underwent unenhanced low-dose chest CT by using either a 64-slice or a 256-slice multi-detector system (GE LightSpeed VCT 64™ and GE Revolution™; General Electric Healthcare); for CT protocol see electronic supplementary material. Lung MRI was performed on a 1.5 Tesla system (Magnetom Symphony fit™; Siemens Healthineers). All patients underwent the following three T1-weighted MRI sequences after application of gadobutrol (Gadovist®, Bayer Healthcare) with a dose of 0.1 mmol kg^−1^ body weight: 2D incoherent (spoiled) GRE, 3D volume interpolated GRE, and 3D UTE sequences. The GRE sequences (both with the Cartesian acquisition of the *k*-space) were performed during breath-hold. The UTE sequence was in-house developed and adapted to lung imaging [28, 29, 32, 33]. The very short echo times of the UTE sequence result in numerous excitations, which makes the sequence robust against motion artefacts [28, 29, 32, 33]. The UTE sequence used 3D radial, centre-out *k*-space trajectories, making it even more robust against motion, flow, and susceptibility artefacts and allowing an acquisition during free-breathing [28, 29, 32, 33]. Image reconstruction for the UTE images was performed in MATLAB (The MathWorks Inc.) [34]. During the lung MRI examinations of the patients, two T2-weighted sequences were also acquired (see electronic supplementary material). The T1-weighted post-contrast sequences were performed in the same order: 2D incoherent (spoiled) GRE, 3D volume interpolated GRE, and 3D UTE sequences. Table 1 shows the standardised technical parameters of the three sequences.

**Table 1:** Technical parameters of the T1-weighted magnetic resonance imaging (MRI) sequences

Parameters	Incoherent GRE	Volume interpolated GRE	UTE
Repetition time (TR) in ms	207.00	3.22	1.10
Echo time (TE) in ms	2.24	1.06	0.08
Flip angle in °	90	10	6
Acquisition matrix	320	320	240
Field of view in mm	350	350	280
Spatial resolution in mm^3^	1.50 × 1.10 × 4.00	1.20 × 1.10 × 4.00	1.17 × 1.17 × 1.17
Slice orientation	Axial	Axial	Axial
Fat saturation	Yes	Yes	Yes
Breath-hold	Yes; acquisition by using four concatenations	Yes, acquisition within one breath-hold	No, acquired during free-breathing with two signal averages
Acquisition time	1.02 min	18.0 s	6.20 min

*GRE*, gradient echo; *UTE*, ultrashort echo time

### Subjective image analysis

All CT and MR images were visually evaluated in consensus by two well-trained radiologists (one general and one paediatric radiologist), each with experience of more than 8 years in chest imaging. The T1-weighted post-contrast MRI sequences and the CT examinations were each separately assessed in an individual blinded session. Subsequently, the reviewers performed a second-look session and compared each of the three MRI sequences with the CT examinations. In this fifth session, the two radiologists assessed whether the pulmonary pathologies could be detected when the CT scans served as a direct comparison. In the case of detection of the pulmonary pathologies in the second-look session, the observers also rated the characteristics of these pathologies.

A structured report was provided for each evaluation session (the imaging evaluation is described in detail in the electronic supplementary material). The image quality of the CT examinations and the three MRI sequences were assessed by considering four criteria: overall image quality, contrast, sharpness, and the presence of artefacts [19, 35, 36]. These imaging parameters were scored on a visual 5-point ordinal Likert scale [19, 35, 36]. The detected pulmonary pathologies, the nodules, and parenchymal areal opacities were described according to current guidelines [37–39]; for details, see the electronic supplementary material.

### Objective image analysis

For quantitative assessment, regions of interests (ROIs) were drawn on the images of the three T1-weighted MRI sequences within the pulmonary lesions (nodules and areal opacities). ROIs with the same size were placed in the lung parenchyma adjacent to the lesions on the same slice. The ROI sizes were chosen as large as possible, while matching the lesions best avoiding vessels, bronchi as well as hilar structures. For each ROI, the mean and the SD of the signal intensity (SI) were recorded. The CNR of the lesions was computed as follows [40, 41]:

$$\mathrm{CNR}=\left({\mathrm{meanSI}}_{\mathrm{lesion}}-{\mathrm{meanSI}}_{\mathrm{parenchyma}}\right)/{\mathrm{SDSI}}_{\mathrm{parenchyma}}$$

### Statistical evaluation

Statistical analysis was performed by using SPSS version 26.0 for Windows (IBM SPSS Statistics). Differences between the image quality parameters of the CT examinations and the MRI sequences were analysed by using Wilcoxon rank sum tests, as the values were not normally distributed according to the results of Shapiro–Wilk tests. McNemar tests evaluated differences in the detection rates of pulmonary nodules and opacities between the MRI sequences. In the case of parameters with normal distribution and interval or ratio scale, paired Student’s *t*-tests were performed to analyse the differences between the CT and the MRI examinations. *p* values < 0.05 (two-sided) were considered in all statistical tests to indicate significance.

## Results

### CT and MR image quality

The results of the image quality assessment are summarised in Table 2. The UTE images scored significantly higher regarding better overall image quality, better sharpness, and fewer artefacts compared to the other T1-weighted sequences. However, the contrast score of UTE imaging was lower with the difference to the incoherent GRE sequence being statistically significant (*p* = 0.002). Comparing image quality parameters of the three T1-weighted MRI sequences between examinations, which were performed with versus without sedation/ anaesthesia and patients aged < 7 years versus ≥ 7 years old, the values were not significantly different (Wilcoxon rank sum tests, *p* > 0.05).

**Table 2:** Image quality parameters of 35 computed tomography (CT) examinations in comparison to the three T1-weighted MRI sequences: incoherent GRE (gradient echo), volume interpolated GRE, and UTE (ultrashort echo time)

Image quality parameter	Chest CT	Incoherent GRE	Volume interpolated GRE	UTE
Mean values of the image quality scores (in parentheses standard deviations)				
Overall image quality	4.4 (0.7)	3.5 (0.8)^†,ǂ^	3.6 (0.9)^†^,^ǂ^	4.1 (0.8)
Contrast	4.7 (0.4)	4.3 (0.5)^†^,^ǂ^	4.1 (0.6)^†^	3.9 (0.6)^†^
Sharpness	4.4 (0.7)	3.5 (0.7)^†^,^ǂ^	3.5 (0.8)^†^,^ǂ^	4.1 (0.9)
Presence of artefacts	4.1 (0.8)	3.1 (0.8)^†^,^ǂ^	3.4 (0.8)^†^,^ǂ^	3.9 (0.8)

Wilcoxon rank sum tests evaluated differences between each of the MRI sequences in comparison to CT examinations as well as between the three MRI sequences

^†^p < 0.05 indicated statistical significance between the MRI sequence and the CT examinations

ǂ p < 0.05 indicated statistical significance between each of the GRE sequences in comparison to UTE images

### Pulmonary nodules

**Fig. 1:**
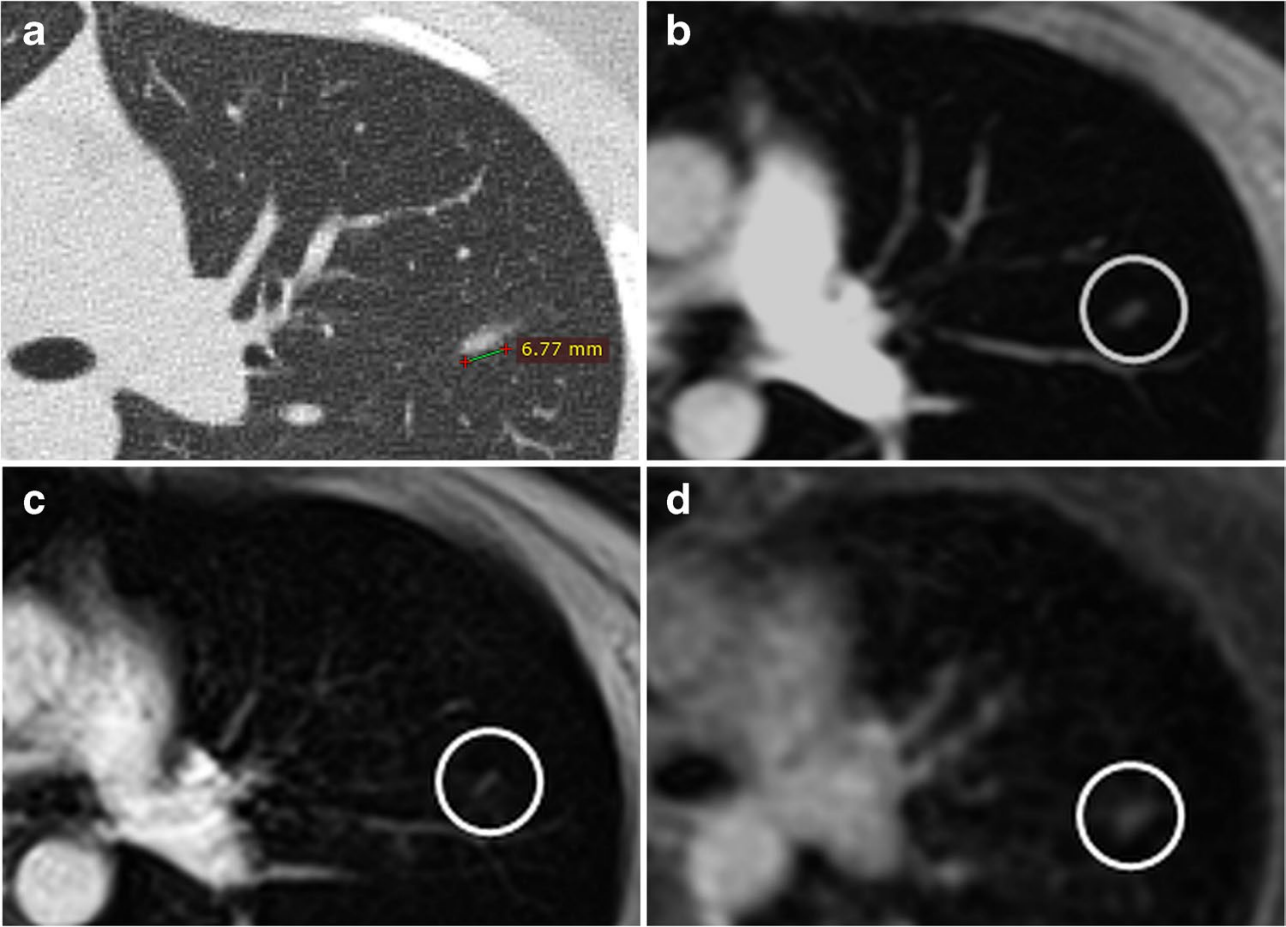
A 10-year-old female patient with rhabdomyosarcoma. An oval-shaped pulmonary nodule with a maximum axial diameter of 7 mm (axial average diameter of 5 mm) was detected on chest CT in the left upper lobe (**a**). The nodule was not detected with the incoherent GRE sequence (**b**) and the volume interpolated GRE sequence (**c**) in the initial blinded session, as the nodule was misinterpreted as part of a vessel due to its linear appearance (circles); furthermore, the lesion was only shown in one image in both GRE sequences due to the slice thicknesses of 4 mm. The oval-shaped nodule (circle) was detected in the UTE image (**d**) in the initial evaluation session; the lesion was additionally seen in 3 images because of the slice thickness of 1.2 mm of the UTE sequence

In the 35 chest CT examinations, 110 pulmonary nodules were detected with a mean of the axial average diameter of 4.9 mm (SD 2.9 mm, range 1–21 mm). The range of the distribution was between 0 (in 15 CT examinations), 1 (in 4), and 31 nodules (in 1) with a mean of 3.1 + / − 6.1 nodules per CT examination. Thus, in 16 CT examinations, at least 2 nodules were detected. A table and a histogram showing the distribution of the 110 nodules within the 35 CT examinations are provided in the electronic supplementary material. The overall detection rate of the 110 nodules, found by CT, was 60.9% (67 of 110 nodules) for incoherent GRE and 62.7% (69 of 110) for volume interpolated GRE sequences; significantly more nodules (84 of 110; 76.4%) were observed in UTE images (McNemar tests, *p* < 0.01). Out of the 4 CT examinations with solely 1 nodule, the UTE technique detected all of the 4 nodules (versus only 1 of 4 nodules was also observed in the incoherent and volume interpolated GRE sequences; axial average diameter 10 mm). The axial average diameters of the 3 only with UTE detected nodules were 4 mm, 5 mm, and 5 mm (Fig. 1). Regarding examinations with ≥ 2 nodules, at least 1 nodule was found in UTE images. Thus, there were no false-negative MRI examinations regarding the UTE technique.

The characteristics of the 110 nodules are described in Table 3. Considering the average diameter of the nodules, more lesions with a size up to 10 mm were found with UTE sequences, whereas the detection rates for nodules with sizes of 1–4 mm differed significantly (see Table 4). Furthermore, nodules, located in the lung periphery, presenting no calcifications, with solid and subsolid appearances as well as with smooth and irregular margins were significantly more often found by UTE imaging (Table 4).

**Table 3:** Characteristics of 110 pulmonary nodules, detected on computed tomography (CT)

Characteristics	
Size group based on the axial average diameter	
1–4 mm	63 (57.3%)
5–7 mm	32 (29.1%)
8–10 mm	11 (10.0%)
> 10 mm	4 (3.6%)
Mediolateral location	
Central	31 (28.2%)
Peripheral	79 (71.8%)
Appearance	
Solid	85 (77.3%)
Subsolid	25 (22.7%)
Margin	
Smooth	60 (54.5%)
Irregular	50 (45.5%)
Presence of calcifications	
Yes	23 (20.9%)
No	87 (79.1%)

**Table 4:** Nodule detection rates in the initial analysis with the MRI sequences incoherent GRE (gradient echo), volume interpolated GRE, and UTE (ultrashort echo time) based on the size and the characteristics of the pulmonary nodules

Detection rates	Incoherent GRE	Volume interpolated GRE	UTE
Size group based on the axial average diameter			
1–4 mm	46.0% (29 of 63)*	49.2% (31 of 63)*	68.3% (43 of 63)*
5–7 mm	78.1% (25 of 32)	78.1% (25 of 32)	84.4% (27 of 32)
8–10 mm	81.8% (9 of 11)	81.8% (9 of 11)	90.9% (10 of 11)
> 10 mm	100.0% (4 of 4)	100.0% (4 of 4)	100.0% (4 of 4)
Mediolateral location			
Central	41.9% (13 of 31)	51.6% (16 of 31)	61.3% (19 of 31)
Peripheral	68.4% (54 of 79)*	67.1% (53 of 79)*	82.3% (65 of 79)*
Appearance			
Solid	67.1% (57 of 85)*	69.4% (59 of 85)*	78.8% (67 of 85)*
Subsolid	40.0% (10 of 25)*	40.0% (10 of 25)*	68.0% (17 of 25)*
Margin			
Smooth	60.0% (36 of 60)*	61.7% (37 of 60)*	75.0% (45 of 60)*
Irregular	62.0% (31 of 50)*	64.0% (32 of 50)*	78.0% (39 of 50)*
Presence of calcifications			
Yes	52.2% (12 of 23)	47.8% (11 of 23)	56.5% (13 of 23)
No	63.2% (55 of 87)*	66.7% (58 of 87)*	81.6% (71 of 87)*

McNemar tests evaluated differences between each of the GRE sequences in comparison to UTE images

^*^p < 0.05 indicated statistical significance

In the second-look session, additional nodules were diagnosed on lung MRI: 15 in incoherent GRE, 12 in volume interpolated GRE, and 11 in UTE images. Seventy-nine pulmonary nodules were seen on chest CT as well as in all three MRI sequences. The average axial diameter of these 79 lesions was as follows: mean size of 5.5 (SD 3.0) mm on CT, 5.2 (SD 3.0) mm in incoherent GRE, 5.3 (SD 3.0) mm in volume interpolated GRE, and 5.3 (SD 3.0) mm in UTE sequences. The size difference between CT and incoherent GRE sequences was statistically significant (paired Student’s *t*-test, *p* = 0.017), between CT and volume interpolated GRE images nearly significant (paired Student’s *t*-test, *p* = 0.052).

In the objective image analysis, the CNR values of the 79 nodules were highest in incoherent GRE sequences with a mean value of 23.7 (SD 13.5), which was significantly higher compared to both, volume interpolated GRE (mean 20.7, SD 13.1; paired Student’s *t*-test, *p* = 0.009) and UTE sequences (mean 19.7, SD 10.9; paired Student’s *t*-test, *p* = 0.013). The mean difference of CNR between volume interpolated GRE and UTE imaging was not significant (paired Student’s t-test, *p* = 0.532).

### Parenchymal areal opacities

**Fig. 2:**
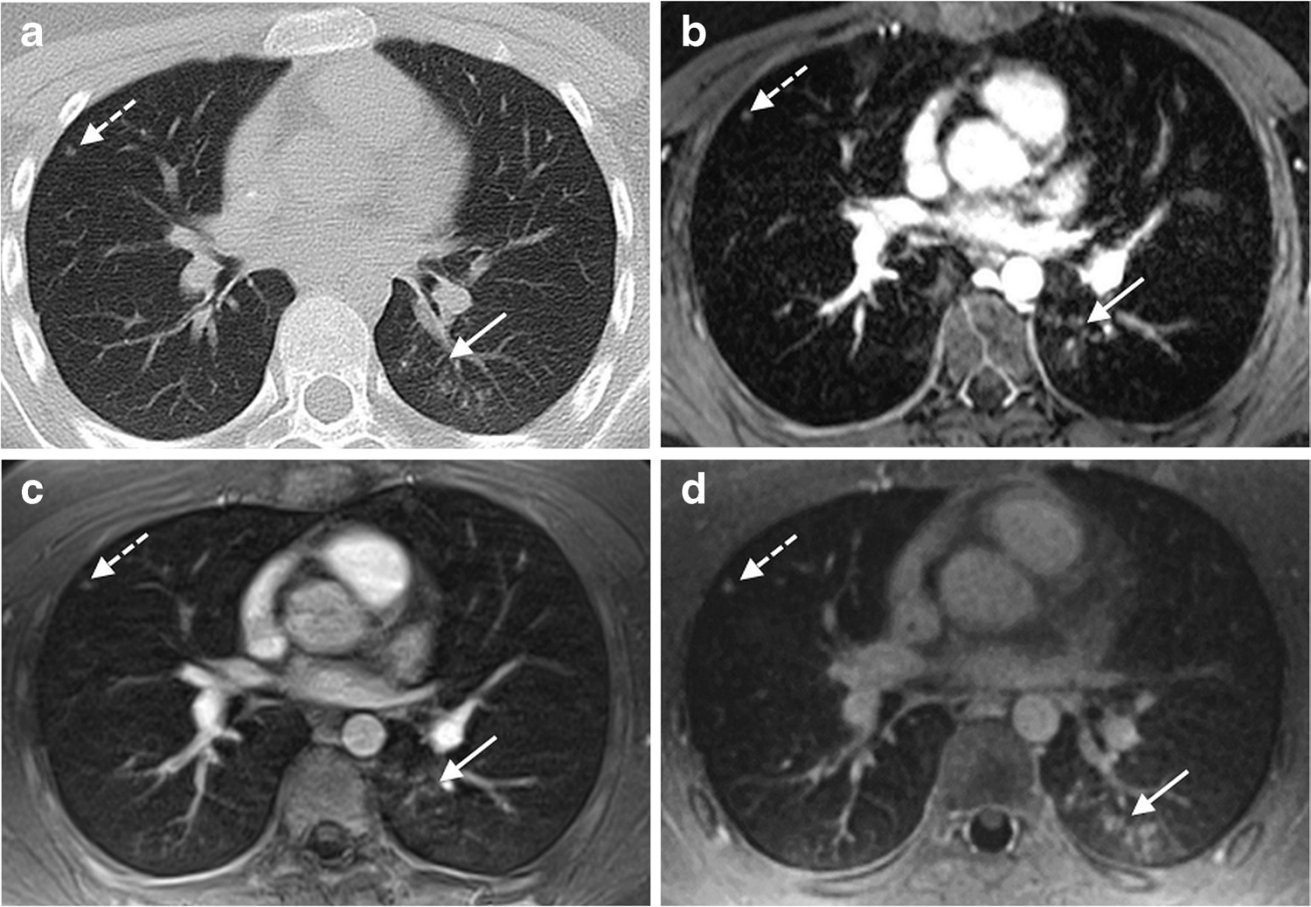
A 14-year-old male patient with Ewing’s sarcoma and aspergillosis. Pulmonary nodule (axial average diameter of 4 mm) in the right middle lobe was detected on CT and on all T1-weighted MR sequences (discontinuous arrows). The aspergillosis infection presented as ground-glass opacity with some nodular pattern in the left lower lobe (continuous arrow). The pathology was diagnosed on chest CT (**a**). With the incoherent GRE (**b**) and the volume interpolated GRE sequence (**c**), the infection was not detected during the initial analysis as it was misinterpreted as hypostasis and artefacts (continuous arrows). However, the ground-glass opacity was observed in the second-look sessions of both sequences in comparison with CT. In the UTE image (**d**), the pathology was correctly diagnosed during the initial evaluation session (continuous arrow)

Forty-five parenchymal areal opacities were diagnosed on chest CT: 16 consolidations, 10 ground-glass opacities, and 19 parenchymal bands. The detection rates in CT and the three MRI sequences are shown in Table 5 (see also Fig. 2). In the second-look session, five additional parenchymal areal opacities were observed with both, incoherent GRE and volume interpolated GRE, and two additional lesions with UTE imaging. Forty pathologies were detected on chest CT as well as with all three MRI sequences in the blinded and/or second-look sessions. The 40 opacities had the following axial average diameters: mean size of 24.1 (SD 17.0) mm on CT, 23.0 (SD 16.5) mm in incoherent GRE, 23.1 (SD 17.4) mm in volume interpolated GRE, and 23.4 (SD 16.8) mm in UTE sequences. The size differences between CT and incoherent GRE (paired Student’s *t*-test, *p* = 0.005) as well as between CT and volume interpolated GRE images (*p* = 0.025) were statistically significant. The size difference between CT and UTE imaging reached a *p* value of 0.088 (paired Student’s *t*-test).

**Table 5:** Detection rates of parenchymal areal opacities in the initial analysis with the MRI sequences incoherent GRE (gradient echo), volume interpolated GRE, and UTE (ultrashort echo time) based on the types of the opacities

Detection rates	Incoherent GRE	Volume interpolated GRE	UTE
Overall parenchymal areal opacities			
	77.8% (35 of 45)*	80.0% (36 of 45)*	93.3% (42 of 45)*
Consolidations			
	100.0% (16 of 16)	100.0% (16 of 16)	100.0% (16 of 16)
Ground-glass opacities			
	50.0% (5 of 10)	60.0% (6 of 10)	90.0% (9 of 10)
Parenchymal bands			
	73.7% (14 of 19)	73.7% (14 of 19)	89.5% (17 of 19)

McNemar tests evaluated differences between each of the GRE sequences in comparison to UTE images

^*^p < 0.05 indicated statistical significance

As in the case of pulmonary nodules, the CNR of the 40 opacities, visible in all three MRI sequences, was highest in incoherent GRE sequence with a value of 28.3 (SD 19.0) followed by UTE (mean 25.8, SD 16.3) and volume interpolated GRE sequences (mean 24.4, SD 15.7) in the objective image analysis. The mean difference between incoherent GRE and UTE images was not significant (paired Student’s *t*-test, *p* = 0.247), the comparison between the GRE sequences was nearly significant (paired Student’s *t*-test, *p* = 0.067). The mean difference of CNR between volume interpolated GRE and UTE imaging was not significant (paired Student’s *t*-test, *p* = 0.480).

## Discussion

Thin-section UTE imaging is a sensitive technique to detect pulmonary nodules and parenchymal areal opacities in children and adolescents with malignancies. To our knowledge, this is the first study, which compared the UTE sequence with two T1-weighted standard sequences, mostly performed in clinical routine, and to the still gold standard chest CT in a paediatric oncologic population. The image quality parameters of the UTE sequence are higher compared to both GRE sequences, except for the contrast.

The high contrast of the incoherent GRE sequence is a direct consequence of the scan parameters, resulting in high signal even from tissues with longer T1-relaxation times. The ultrashort echo time of the UTE sequence leads to substantial signal gain in the lung parenchyma [27, 42]. Nevertheless, despite this reduction of contrast, which could be mitigated by increasing the TR (albeit at the cost of scan time), the nodular detection rate of UTE was superior compared to the other two MRI sequences, mainly due to the reduced artefacts and the improved lesion sharpness (due to the high isotropic resolution). The mean overall image quality of 4.1 for the UTE sequence in our study is comparable with published findings in adults. Ohno et al. [43] reported a mean overall image quality of 4.4 with 1 mm^3^ isotropic UTE imaging, using a similar visual 5-point Likert scale. In the study of Cha et al. [36], the UTE sequence resulted in a mean overall image quality of 3.9–4.0 and a mean value of 3.7 for the presence of image artefacts, scored by two readers with a similar 5-point Likert scale.

The significantly higher detection of pulmonary nodules with UTE sequence in our study is also similar to published reports in adults, which compared MRI sequences to chest CT. In the study of Burris et al. [12], the overall detection rate was 73% (60 of 82 nodules) for UTE and 30% (25 of 82 nodules) for dual-echo GRE sequences, whereby the nodules had a mean short-axis diameter of 6.2 mm. Wielpütz et al. [39] reported a detection rate of the UTE sequence of 92.5% for nodules with a relatively high mean maximum long-axis diameter of 17.4 mm. In our investigation, the UTE sequence was most beneficial, compared to GRE images, in diagnosing challenging nodules with sizes up to 4 mm. In the study of Burris et al. [12], the higher detection of the UTE sequence was statistically significant for nodules with diameters ≥ 4 mm, but not for sizes ≥ 8 mm. We detected significantly more solid and subsolid nodules with the UTE sequence compared to the GRE images. Ohno et al. [44] evaluated in adults the performance of UTE imaging compared to chest CT depending on the nodular appearance and found that 35.3% ground-glass, 33.3% partly solid, and 94.7% solid nodules (long-axis diameters of 4–6 mm) were detected with the UTE technique in consensus reading. The UTE sequence is also suited in diagnosing subsolid nodules, which are diagnostically challenging. This higher detection in our study is most likely due to the combination of higher spatial resolution and higher lung signal of the UTE technique. However, the UTE sequence showed no significant benefit in detecting calcified nodules in our study. Calcified nodules remain a diagnostic challenge for MRI due to their low signal intensity [4, 17, 45]. Although most calcified pulmonary nodules are likely to be benign, some bone-forming paediatric tumours, e.g., osteosarcomas, can show metastases with at least some calcifications in the lungs [4, 17, 45].

As described, the UTE sequence missed no patients with pulmonary nodules; out of the 4 CT examinations with solely 1 nodule, the UTE technique detected all of the 4 nodules in the initial reading. Thus, the missing of some nodules by UTE did not have a significant impact on the treatment of the patients. Furthermore, 7 of the 15 in total missed nodules could be visualised on MRI by considering all sequences (with also T2-weighted images). Although the radiation doses of chest CT examinations are quite low by using new technologies, any radiation exposure should be avoided in children if radiation-sensitive organs, e.g., thyroid gland, breasts, red bone marrow, are located in the exposure fields and in the case of tumour predisposition syndromes [2]. The application of contrast media may be favourable for diagnosing mediastinal and bihilar lymph node metastases on MR and CT imaging, whereas enhanced CT examinations result in higher radiation exposures than unenhanced CT imaging. Another advantage of MRI is the distinct diagnosis of bone metastases [3, 5, 45]. As no breath-hold manoeuvres are necessary for the performance of the UTE sequence, the higher detection rates and the possible acquisition without intubation anaesthesia compensate for the higher acquisition time of the UTE sequence compared to the common standard T1-weighted GRE sequences.

Lung MRI is increasingly performed in children for diagnosing pulmonary infections [46, 47]. All consolidations were detected with all three MRI sequences in our study, in contrast to the more subtle ground-glass opacities and parenchymal bands. Due to the low number of these pathologies, the higher detection rate with UTE compared to the GRE images was statistically not significant. The diagnostic challenge of detecting ground-glass opacities by MRI has been reported. Sodhi et al. [46] evaluated an MRI protocol with T2-weighted and volume interpolated GRE sequences and found a high correlation with chest CT for the diagnosis of consolidations, but not for ground-glass opacities in children (age range 5–15 years). Roach et al. [48] compared UTE imaging (acquisition time 5–10 min) with chest CT in patients with early cystic fibrosis lung disease (mean age 31.8 months) and reported Pearson correlation coefficients of 0.45 for diagnosing consolidations and of 0.21 for diagnosing ground-glass opacities. In older patients with cystic fibrosis (mean age 22.6 years), Dournes et al. [49] found that their evaluated UTE sequence showed good to very good agreement with CT regarding all morphologic pulmonary manifestations, such as bronchiectasis, peribronchial thickening, and mosaic pattern. Thus, the UTE technique becomes increasingly important for the diagnosis of subtle parenchymal pathologies.

Since the inclusion criteria required a homogeneous study design, only a limited number of patients could be included in this prospective study. All patients had clinical indications for the administration of MR contrast media; no agent was applied only for the study purpose. The use of contrast medium might have had an impact on the diagnostic performance, as the detection of pulmonary nodules can be improved by possible enhancement [10, 40]. However, MR contrast media must be in general administered judiciously in children and adolescents, due to possible gadolinium deposition in different tissues, such as brain, bone, or liver, and the brain development during childhood and adolescence [50]. The evaluated sequences were acquired at the end of the MRI examinations; thus, image artefacts due to patient movements or less deep anaesthesia may have occurred. Furthermore, a wash-out of the contrast media might have developed. The diagnostic performance of the T1-weighted sequences, particularly the UTE technique, which was performed as the last sequence, could be even higher if they were acquired earlier in the MRI examinations.

In conclusion, the findings of our study indicate that UTE imaging of the lung leads to improved diagnostic performance compared to conventional T1-weighted sequences in children and adolescents, if chest CT served as the gold standard. In particular, the detection rate of small pulmonary nodules ≤ 4 mm is superior with UTE imaging, presumably due to the higher spatial resolution and reduced artefacts. The visibility of subtle, diffuse parenchymal lesions, e.g., ground-glass opacities, in UTE images is promising. Lung MRI may be used instead of chest CT examinations during the staging of children with oncologic diseases, particularly if the diagnosis of lymph node and bone metastases are also important issues.

## Supplementary Material

ESM 1 (pdf 145 kb)

## Abbreviations

2D: Two-dimensional
3D: Three-dimensional
CNR: Contrast-to-noise ratio
CT: Computed tomography
GRE: Gradient echo
MRI: Magnetic resonance imaging
SD: Standard deviation
SI: Signal intensity
TE: Echo time
TR: Repetition time
UTE: Ultrashort echo time

## Declarations

### Guarantor

The scientific guarantor of this publication is Prof. Dr. Diane M. Renz, M.D.

### Statistics and biometry

Some of the authors have significant statistical expertise. No complex statistical methods were necessary for this paper.

### Methodology

• prospective

• cross-sectional study

• performed at one institution
