# Supplementary material for: Ultrashort echo time MRI of the lung in children and adolescents: comparison with non-enhanced computed tomography and standard post-contrast T1w MRI sequences
Source: Eur Radiol. 2021 Oct 20;32(3):1833–42. doi: 10.1007/s00330-021-08236-7 (PMC8831263; doi:10.1007/s00330-021-08236-7)
Supplement: Supplementary file 1 — (pdf 145 kb) [file 330_2021_8236_MOESM1_ESM.pdf]

<u>**Supplementary material to the materials and methods of the article:**</u>

Ultrashort echo time MRI of the lung in children and adolescents: comparison with non-enhanced computed tomography and standard post-contrast T1w MRI sequences

Computed tomography (CT) protocol

The following standardized technical CT parameters were used: axial slice orientation, inspiratory breath-hold, tube voltage 80 kVp, dose-modulated tube current (10-80 mAs), pitch 1.375, section thickness 0.625 mm, rotation time 0.3-0.5 s, 40% ASIR (adaptive statistical iterative reconstruction), no application of contrast medium. The chest CT examinations had a mean volumetric CT dose index ($CTDI_{vol}$) of 0.72 +/- 0.30 mGy, a mean dose length product (DLP) of 17.3 +/- 5.0 mGy cm, and a mean effective dose of 0.26 +/- 0.07 mSv.

Magnetic resonance imaging (MRI) protocol

During the lung MRI examinations of the patients, two T2-weighted sequences were also acquired: echo-planar fast spin echo and turbo spin echo sequence. The technical parameters of the T2-weighted magnetic resonance imaging (MRI) sequences were as follows:

Parameters	Echo-planar fast spin echo	Turbo spin echo
Repetition time (TR) in ms	600	1810
Echo time (TE) in ms	72	108
Flip angle in °	150	136
Acquisition matrix	320	320
Field of view in mm	380	350
Spatial resolution in mm^3	1.5 x 1.2 x 4.0	1.1 x 1.1 x 4.0
Slice orientation	Coronal	Axial
Fat saturation	Yes	No
Breath-hold	Yes; acquisition by using three concatenations	No, acquisition by using respiratory navigator triggering
Acquisition time	30.0 s	Depending on the navigator triggering; the mean acquisition time was 6.5 min (+/- 2.8 min)

Unenhanced 3D volume interpolated GRE T1-weighted sequence was also performed before the application of contrast media (acquisition time 18.0 s; technical parameters see Table 1 of the manuscript). The mean duration of the whole lung MRI examination was 14.9 +/- 3.2 min.

Analysis of images and pulmonary pathologies

A structured report was provided for the evaluation sessions. The image quality of the computed tomography examinations and the three magnetic resonance imaging sequences were assessed by considering four criteria: overall image quality, contrast, sharpness, and the presence of artefacts [1-3]. The imaging parameters were scored on a visual 5-point ordinal Likert scale with the following categories [1-3]:

- 1 = unacceptable (overall image quality, contrast); unreadable (sharpness, artefacts);
- 2 = poor (overall image quality, contrast); extreme blur (sharpness); extreme artefacts (artefacts);
- 3 = acceptable (overall image quality, contrast); moderate blur (sharpness); moderate artefacts (artefacts);
- 4 = good (overall image quality, contrast); mild blur (sharpness); mild artefacts (artefacts);
- 5 = excellent (overall image quality, contrast); no blur (sharpness); no artefacts (artefacts).

The detected pulmonary pathologies were described according to current guidelines [4-6]:

- **Pulmonary nodules:**

  A *solitary pulmonary nodule* is defined as a rounded opacity which is at least moderately well marginated and no larger than 3 cm in its maximum diameter. The pulmonary nodules were rated in the computed tomography (CT) examinations by using the following features:

  - *solid* versus *subsolid appearance* (with at least parts of ground glass opacities);

  - *smooth* versus *irregular margin*;

  - *presence of calcifications* versus *no detection of calcifications*.

- **Parenchymal areal opacities:**

  Areas, which appear more opaque compared to the surrounding lung parenchyma, were categorized into three different pathologies:

  - *consolidation:* an area of increased parenchymal attenuation, which obscures the margins of vessels and airway walls;

  - *ground-glass opacity:* an area of increased parenchymal attenuation with preservation of bronchial and vascular margins;

  - *parenchymal band:* a linear opacity, usually 1-3 mm thick and up to 5 cm long, that usually extends to the visceral pleura and reflects pleuroparenchymal fibrosis, e.g., scar post-surgery.

Within the description, the *mediolateral localization* of pulmonary nodules was also assessed. Nodules were considered as *peripheral* if they were detected within 2 cm of the chest wall; all other lesions were characterized to be in *central* localization [7]. The axial diameter of a pulmonary pathology (nodule and opacity) was determined as the average of its maximum long-axis and its perpendicular maximum short-axis measurement, conducted in the same plane, according to current recommendations [8]. Nodules were categorized into four subdivisions based on their average diameter: 1-4 mm, 5-7 mm, 8-10 mm, and >10 mm [7, 9].

All CT and MR images were visually evaluated in consensus by two well-trained radiologists (one general and one paediatric radiologist), each with experience of more than eight years in chest imaging. To determine the diagnostic performance of the T1-weighted post-contrast MRI sequences, they were each separately assessed in three individual sessions. The CT examinations were reviewed in a fourth session and served as the diagnostic gold standard. All CT and MR images were evaluated in a random order, and the readers were blinded to clinical and previous radiological data to ensure reliable unbiased imaging comparison. Subsequently, the reviewers performed a second-look session and compared each of the three MRI sequences with the CT examinations. In this fifth session, the two radiologists assessed whether the pulmonary pathologies could be detected when the CT scans served as direct comparison. In the case of detection of the pulmonary pathologies in the second-look session, the observers also rated the characteristics of the pathologies. The time interval between each of the five reading sessions was at least three weeks to avoid recall bias.

Supplementary material to the study results of the article:

Ultrashort echo time MRI of the lung in children and adolescents: comparison with non-enhanced computed tomography and standard post-contrast T1w MRI sequences

The table and the histogram show the distribution of the 110 pulmonary nodules within the 35 CT examinations:

Number of nodules	Number of CT examinations	Percentages in %
0	15	42.9
1	4	11.4
2	5	14.3
3	4	11.4
8	1	2.9
13	1	2.9
17	1	2.9
31	1	2.9
	35	100.0

Number of pulmonary nodules

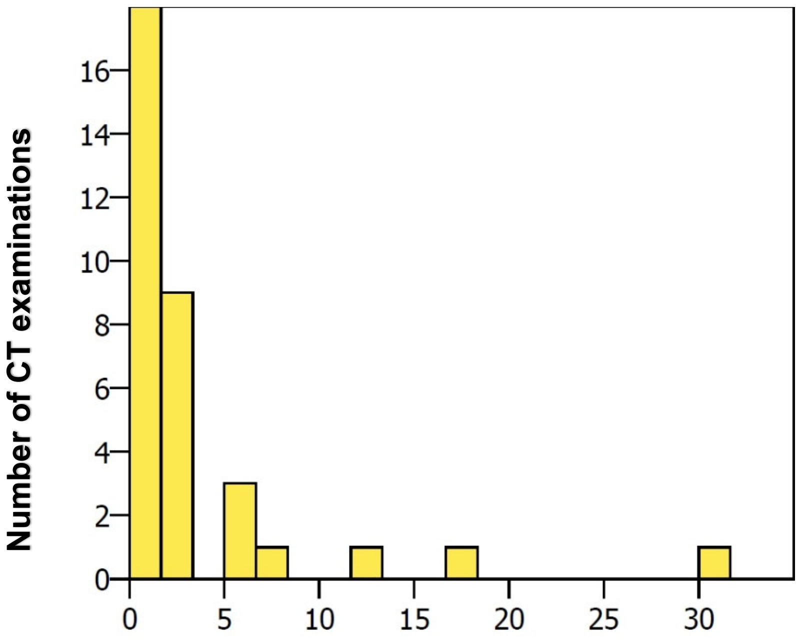

References

1. Scholz O, Denecke T, Böttcher J et al (2017) MRI of cystic fibrosis lung manifestations: sequence evaluation and clinical outcome analysis. Clin Radiol 72:754-763

2. Pierre EY, Grodzki D, Aandal G et al (2014) Parallel imaging-based reduction of acoustic noise for clinical magnetic resonance imaging. Invest Radiol 49:620-626

3. Cha MJ, Park HJ, Paek MY et al (2018) Free-breathing ultrashort echo time lung magnetic resonance imaging using stack-of-spirals acquisition: a feasibility study in oncology patients. Magn Reson Imaging 51:137-143

4. Hansell DM, Bankier AA, MacMahon H, McLoud TC, Müller NL, Remy J (2008) Fleischner Society: glossary of terms for thoracic imaging. Radiology 246:697-722

5. Truong MT, Ko JP, Rossi SE et al (2014) Update in the evaluation of the solitary pulmonary nodule. Radiographics 34:1658-1679

6. Wielpütz MO, Lee HY, Koyama H et al (2018) Morphologic characterization of pulmonary nodules with ultrashort TE MRI at 3T. AJR Am J Roentgenol 210:1216-1225

7. Burris NS, Johnson KM, Larson PE et al (2016) Detection of small pulmonary nodules with ultrashort echo time sequences in oncology patients by using a PET/MR system. Radiology 278:239-246

8. Bankier AA, MacMahon H, Goo JM, Rubin GD, Schaefer-Prokop CM, Naidich DP (2017) Recommendations for measuring pulmonary nodules at CT: a statement from the Fleischner society. Radiology 285:584-600

9. Nagel SN, Wyschkon S, Schwartz S, Hamm B, Elgeti T (2016) Can magnetic resonance imaging be an alternative to computed tomography in immunocompromised patients with suspected fungal infections? Feasibility of a speed optimized examination protocol at 3 Tesla. Eur J Radiol 85:857-863
